# Effect of Supplementation with *Lactobacillus reuteri* SGL 01 in Lactating Women on Breast Milk and Neonatal Gut Microbiota: An Exploratory, Randomized, Open-Label Clinical Trial

**DOI:** 10.3390/nu18050794

**Published:** 2026-02-28

**Authors:** Elia Pagliarini, Caterina Poli, Silvia Martini, Anna Giulia Cimatti, Diana Di Gioia, Luigi Tommaso Corvaglia

**Affiliations:** 1 Department of Agricultural Sciences, University of Bologna, Viale Fanin 42, 40127 Bologna, Italy; elia.pagliarini2@unibo.it (E.P.); diana.digioia@unibo.it (D.D.G.); 2 Neonatal Intensive Care Unit, IRCCS AOUBO, 40138 Bologna, Italy; caterina.poli4@studio.unibo.it (C.P.); agiulia.cimatti@hotmail.it (A.G.C.); luigi.corvaglia@unibo.it (L.T.C.); 3 Department of Medical and Surgical Sciences, University of Bologna, 40138 Bologna, Italy

**Keywords:** nutritional supplements, probiotics, *Lactobacillus reuteri*, gut microbiota, neonates, breast milk, breastfeeding, fecal samples, qPCR

## Abstract

**Background/Objectives**: Early-life gut colonization is crucial for immune system development and metabolic programming. *Lactobacillus reuteri* has been investigated for its capacity to modulate neonatal gut microbiota, but evidence regarding maternal supplementation during lactation remains limited. This study aimed to evaluate the effect of maternal supplementation with *L. reuteri* SGL 01 on the microbial composition of breast milk and neonatal feces over the first month of life. **Methods**: This is an exploratory, prospective, open-label randomized clinical trial. Lactating mothers of full-term and exclusively breastfed infants were randomized to receive either daily *L. reuteri* SGL 01 (1 × 10^9^ CFU) for 30 days or no supplementation. Quantitative real-time PCR was used to assess *Bifidobacterium* spp., *Lactobacillus* spp., *Clostridium* spp., and the *Bacteroides fragilis* group in maternal milk and neonatal feces at baseline (T0) and after 30 days (T1). **Results**: Twenty-seven mother–infant dyads completed the study (15 supplemented, 12 controls). No significant changes in breast milk microbiota composition were observed across any of the bacterial taxa following maternal supplementation. In contrast, neonatal fecal samples from the supplemented group showed significant increases in *Bifidobacterium* spp. (*p* < 0.001), *Lactobacillus* spp. (*p* = 0.029), and *Clostridium* spp. (*p* = 0.003) at T1. No significant microbial changes were observed in the control group, except for a slight reduction in *Clostridium* spp. (*p* = 0.046). **Conclusions**: Maternal supplementation with *L. reuteri* SGL 01 did not modify breast milk microbiota but was associated with a modulation of neonatal gut colonization, including an increased abundance of beneficial taxa such as *Bifidobacterium*, suggesting potential indirect maternal-to-infant microbial effects.

## 1. Introduction

Early-life gut colonization is a critical process that plays a pivotal role in immune system maturation, metabolic programming, and protection against pathogenic microorganisms [1,2]. The neonatal period represents a highly plastic developmental window during which environmental and maternal factors, including mode of delivery, feeding practices, antibiotic exposure, and maternal microbiota, can profoundly influence microbial succession and assembly, with potential long-term consequences for health outcomes [3,4]. Perturbations in early microbial colonization have been associated with an increased risk of immune-mediated and metabolic disorders, including allergic diseases, autoimmune conditions, obesity, and gastrointestinal dysfunction [5,6].

Probiotic supplementation has therefore emerged as a promising strategy to modulate the neonatal gut microbiota. *Lactobacillus reuteri* (*L. reuteri*) has been shown to enhance gut microbial diversity, reduce the abundance of potentially pathogenic taxa such as *Enterobacteriaceae* and *Staphylococcaceae*, and promote the establishment of beneficial commensal microorganisms, particularly in preterm infants [7]. However, these effects are often transient and may require sustained or repeated administration to support long-term microbial stability [3]. Beyond its role in microbial modulation, *L. reuteri* has demonstrated therapeutic efficacy in the management of infantile colic. Clinical studies suggest that its anti-inflammatory and immunomodulatory properties may contribute to reductions in crying duration among breastfed infants [8,9]. Collectively, these findings underscore the dual role of *L. reuteri* in shaping gut microbiota composition and improving functional gastrointestinal outcomes.

Human milk is a complex bioactive fluid containing a dynamic microbiota that plays a crucial role in shaping the neonatal gut microbiome [10]. Although the milk microbiota was initially thought to originate primarily from maternal skin or the infant’s oral cavity, growing evidence supports the existence of an entero-mammary pathway, through which maternal gut–derived bacteria translocate to the mammary gland via immune cells, such as dendritic cells and macrophages [11,12,13]. This vertical transmission route provides a plausible mechanism by which maternal interventions may influence the infant’s gut microbiota.

Despite the well-established benefits of direct neonatal probiotic supplementation, the effects of maternal *L. reuteri* intake during lactation on the microbiota composition of human breast milk and, consequently, on neonatal gut colonization, remain poorly investigated. This approach, which could also be beneficial to the maternal microbiome, may serve as a practical alternative to direct neonatal probiotic administration. Available evidence is largely derived from animal models, in which maternal administration of *L. reuteri* has been associated with favorable modulation of both colostrum and neonatal gut microbiota [14,15].

The present study aimed to evaluate whether maternal supplementation with *Lactobacillus reuteri* SGL 01 during the first postpartum month in mothers of full-term, exclusively breastfed infants could modify the microbial composition of breast milk and the intestinal microbiota of their infants.

## 2. Materials and Methods

### 2.1. Study Design and Ethics

This spontaneous, exploratory, prospective, single-center, open-label interventional study was conducted at the Postnatal Unit of the IRCCS Azienda Ospedaliero-Universitaria di Bologna (AOUBO), Italy, between October 2017 and January 2021. The study protocol was approved by the local Ethics Committee and was conducted in accordance with Good Clinical Practice guidelines and the principles of the Declaration of Helsinki. Written informed consent was obtained from all participants.

Eligible participants were Caucasian women aged ≥18 years admitted to the local Obstetric/Postnatal Unit who had vaginally delivered full-term infants and were exclusively breastfeeding. Exclusion criteria included maternal pharmacological treatments and medical conditions contraindicating breastfeeding or probiotic supplementation.

Participants were block-randomized in a 1:1 ratio to either the interventional or non-interventional group within 48 h after delivery. Women who provided informed consent to participate in the study were randomly assigned, in an open-label design, to either the intervention group (Group 1) or the control group (Group 2). Participants allocated to Group 1 received daily oral supplementation with *L. reuteri* SGL 01 [16]. The probiotic was administered once daily as five oral drops, delivering approximately 1 × 10^9^ colony-forming units (CFU) of *L. reuteri* SGL 01, for 30 consecutive days, starting within the first postpartum week. Participants allocated to Group 2 received no supplementation and served as controls.

### 2.2. Sample Collection

In both groups, breast milk samples were collected at two time points: prior to the initiation of supplementation (T0) and after 30 consecutive days (T1).

Breast milk was manually expressed either by midwives (when mothers were still hospitalized) or by the mothers themselves. The first drops of milk were discarded, after which approximately 5–10 mL of midstream milk was collected into sterile 15 mL tubes (Merck KGaA, Darmstadt, Germany). Approximately 2–5 g of stool samples were transferred into sterile 50 mL tubes using sterile spatulas.

Breast milk and stool samples were immediately frozen at −20 °C and transported to the research facility within 24 h in insulated containers with frozen gel packs. Upon arrival, samples were stored at −80 °C until analysis. DNA extraction was performed within one week of sample collection.

### 2.3. Microbial Composition Analysis

Targeted microbial groups, selected for their functional role in newborns (*Bifidobacterium* spp., *Lactobacillus* spp., *Bacteroides fragilis* group including *B. fragilis*, *B. distasonis*, *B. ovatus*, *B. thetaiotaomicron*, *B. vulgatus*, and *Clostridium* spp.), were quantified using real-time PCR performed on both milk and fecal samples. Analyses were performed at the Department of Agricultural and Food Sciences of the University of Bologna by personnel blinded to maternal randomization.

The selected taxa represent key functional groups involved in early gut colonization: *Bifidobacterium* spp. and *Lactobacillus* spp. are recognized as beneficial, breast milk-associated colonizers [16]; the *Bacteroides fragilis* group represents components of an adult-like microbiota emerging during the first month of life; and *Clostridium* spp. play an important role in gut maturation. *Clostridium* and *Bacteroides* were analyzed exclusively in infant feces, as these anaerobic taxa are not core members of the breast milk microbiota [17,18].

Bacterial DNA was extracted from 250 mg of fecal material and 2 mL of milk samples, stored at −80 °C after collection, using the QIAamp DNA Stool Mini Kit (Qiagen, Hilden, Germany) with minor modifications to the manufacturer’s protocol. Specifically, an additional incubation step at 95 °C for 10 min followed by 2 cycles at −80 °C was introduced in the presence of lysis buffer to enhance bacterial cell disruption in both stool and milk samples. Extracted DNA was stored at −80 °C until further analysis.

The purity of the extracted DNA was assessed by measuring the absorbance ratio at 260/280 nm using an Infinite^®^ 200 PRO NanoQuant spectrophotometer (Tecan, Männedorf, Switzerland). DNA concentration was determined with a Qubit^®^ 3.0 Fluorometer (Invitrogen, Life Technologies, Carlsbad, CA, USA). Quantification of selected microbial groups was performed using 20 µL PCR reactions containing 10 µL of Fast SYBR^®^ Green Master Mix (Applied Biosystems, Foster City, CA, USA), optimized primer concentrations (Table 1 and Table 2), molecular-grade water and 2 µL of DNA template (2.5 ng/µL). Primer concentrations were optimized using primer matrix tests on 48-well plates to determine the optimal Ct/Rn ratio [19].

The obtained cycle threshold values were transformed into bacterial counts (log CFU/g feces and log CFU/mL milk) based on rRNA gene copy numbers retrieved from the rRNA copy number database [20]. Standard curves were generated using 16S rRNA gene PCR products from type strains of each target microorganism. PCR products were purified using the NucleoSpin^®^ Extract II kit (MACHEREY-NAGEL GmbH & Co. KG, Düren, Germany), quantified at 260 nm, and serially diluted to obtain 10^2^, 10^3^, 10^4^, 10^5^, 10^6^, and 10^7^ gene copies per reaction for calibration.

Although the study adopted an open-label design for probiotic supplementation, all microbiological analyses were performed by laboratory personnel blinded to maternal randomization group allocation. Samples were coded with unique identifiers prior to analysis, and investigators performing DNA extraction and quantitative polymerase chain reaction (qPCR) assays had no access to clinical or group assignment information. This approach ensured an unbiased assessment of microbial outcomes despite the open-label nature of the intervention.

### 2.4. Statistical Analysis

IBM SPSS Statistics version 28 (Statistical Package for the Social Sciences; SPSS Inc., Chicago, IL, USA) was used for statistical analysis. Patients with missing data at T1 were excluded from the analysis. Mann–Whitney U test was used to compare the concentration of *Lactobacillus*, *Bifidobacterium*, *Clostridium* and *Bacteroides* spp. in milk and fecal samples at T0 and T1 between the study groups. The Wilcoxon signed rank test was used to compare the concentration of *Lactobacillus*, *Bifidobacterium*, *Clostridium* and *Bacteroides* spp. in milk and fecal samples between T0 and T1 within each study group. Significance level was set at *p* < 0.05.

## 3. Results

A total of 70 healthy mother-infant dyads, all delivered by spontaneous vaginal birth and exclusively breastfed, were enrolled in the study and allocated to the two study groups according to maternal supplementation with *L. reuteri*.

**Figure 1:**
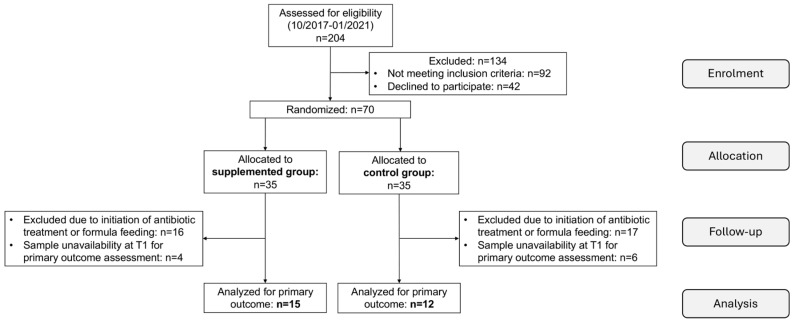
CONSORT flow diagram of the progress through the study phases.

As shown in the enrolment flow chart (Figure 1), 43 dyads were ruled out from the study analysis after enrollment for the following reasons: unavailability of stool or milk samples at T1 (n = 10), initiation of antibiotic therapy or introduction of formula feeding (n = 33). The intervention group ultimately comprised 15 mothers who received daily probiotic supplementation and their neonates, whereas 12 maternal-neonatal dyads from the non-supplemented control group were included in the final analysis.

All participants resided in the metropolitan area of Bologna, Italy. Median maternal age, pre-pregnancy body mass index (BMI), smoking habits during pregnancy, BMI and gestational age at birth and neonatal birth weight in the supplemented and control groups are shown in Table 3; no significant between-group differences were observed. Supplementation was well-tolerated by all the supplemented mothers and no adverse effects were reported.

Concentrations of *Lactobacillus* and *Bifidobacterium* spp. in maternal milk samples and neonatal fecal samples from both study groups are illustrated in Table 4 and Table 5, respectively, whereas neonatal fecal concentrations of *Clostridium* and *Bacteroides* spp. are provided in Table 6. Significant comparisons between T0 and T1 are highlighted in bold.

In the supplemented group (milk samples, n = 15; fecal samples, n = 15), no significant differences were observed in the concentration of both *Lactobacillus* and *Bifidobacterium* spp. in maternal milk between T0 and T1. In contrast, neonatal fecal samples showed a significant increase in both *Bifidobacterium* spp. (*p* < 0.001) and *Lactobacillus* spp. (*p* = 0.029) at T1. *Clostridium* and *Bacteroides* counts were analyzed in 14 out of 15 fecal samples from the supplemented group: while a significant increase in *Clostridium* spp. was observed at T1 (*p* = 0.003), *Bacteroides* spp. remained unchanged.

In the non-supplemented control group, no significant changes in *Bifidobacterium* spp. and *Lactobacillus* spp. were observed between T0 and T1 in maternal milk samples (n = 12) and neonatal fecal samples (n = 8). *Clostridium* spp. and *Bacteroides* spp. were analyzed in 7 out of 8 fecal samples from the control group, showing a slight but statistically significant reduction in *Clostridium* spp. (*p* = 0.046), whereas no significant changes were detected in *Bacteroides* spp.

No significant between-group difference was observed in the concentration of *Lactobacillus*, *Bifidobacterium*, *Clostridium* and *Bacteroides* spp. in milk and fecal samples at both T0 and T1.

## 4. Discussion

This study examined the effects of maternal supplementation with *L. reuteri* SGL 01 during the first month of lactation on the microbial composition of breast milk and on the fecal microbiota of exclusively breastfed neonates. A quantitative assessment of key bacterial groups' characteristics of early-life microbiota was performed using a targeted molecular approach focusing on *Bifidobacterium* spp., *Lactobacillus* spp., *Bacteroides fragilis* group spp. and *Clostridium* spp. [21,22].

Analysis of breast milk samples revealed stable levels of *Bifidobacterium* spp. and *Lactobacillus* spp. over time in both supplemented and non-supplemented groups. These findings are consistent with previous reports suggesting that the core bacterial composition of human milk exhibits limited short-term variability and is predominantly influenced by host-related factors, stage of lactation and intrinsic milk components [23,24]. Such stability supports the concept that the human milk microbiota is relatively resilient to short-term dietary or probiotic interventions, particularly when these do not induce systemic effects capable of significantly influencing entero-mammary immune cell trafficking. Moreover, this apparent resilience may reflect tightly regulated host–microbe interactions within the mammary gland, which contribute to maintaining a conserved microbial profile throughout lactation. The absence of detectable changes may also be partially explained by the low relative abundance of probiotic-derived taxa in breast milk, which may remain below the sensitivity threshold of qPCR-based detection methodologies [25]. Overall, these findings suggest that short-term interventions may have limited effects on the dominant milk-associated microbial populations, underscoring the need for longer intervention periods or complementary high-resolution analytical approaches to capture subtle or transient microbial shifts.

Neonatal fecal samples from the supplemented group exhibited a significant increase in *Bifidobacterium* spp. and *Lactobacillus* spp. after 30 days of life. These taxa play a key role in the early gut colonization and are strongly promoted by breastfeeding and by specific metabolic substrates, such as human milk oligosaccharides [26]. The observed increase suggests that maternal supplementation with *L. reuteri* SGL 01 may indirectly modulate the infant gut ecosystem, even in the absence of detectable changes in breast milk microbiota. Similar findings have been reported following other maternal probiotic interventions, supporting the involvement of systemic or immunological mechanisms such as modulation of milk cytokines, secretory IgA, or antimicrobial peptides that may enhance neonatal gut colonization by beneficial taxa [27].

The increase detected in the supplemented group aligns with the concept of maternal–infant microbial interplay, which may operate through multiple biological and non-biological pathways, including the transfer of low-abundance maternal strains and the modulation of milk-derived bioactive factors [28]. An alternative hypothesis is that maternal probiotic intake may exert its effects by shaping the functional properties of breast milk rather than its taxonomic composition. Indeed, increasing evidence indicates that probiotics can influence milk metabolome (e.g., short-chain fatty acids, indole derivatives) and immune-active components, including cytokines and immunoglobulins. These bioactive molecules may indirectly shape the infant gut microbiota by creating a selective ecological niche that favors the expansion of specific microbial groups, even in the absence of detectable compositional changes in milk [29]. Another possible mechanism involves maternal supplementation with *L. reuteri*, which may be transmitted vertically during delivery and/or through early skin-to-skin contact. During vaginal delivery, the newborn is exposed to the maternal vaginal and intestinal microbiota, which colonize the neonatal gut [30]. Furthermore, oral-to-oral transfer during breastfeeding and repeated skin-to-skin contact provide ongoing opportunities for bacterial exchange, independent of the composition of mature milk [30].

Regarding *Clostridium* spp., an increase was observed in the supplemented group at 30 days of life. Early gut colonization by *Clostridium* species represents a recognized stage of neonatal microbial succession, and the increase observed in the present study is consistent with the progressive diversification of the infant gut microbiota during the first month of life [31]. The presence of *Clostridium* species in early infancy reflects the ongoing transition of the gut microbiota toward a more complex and mature community and may indicate ongoing maturation of the gut microbial network. The stability of the *Bacteroides fragilis* group over time is consistent with previous studies reporting individualized trajectories of *Bacteroides* colonization influenced by delivery mode, feeding type, and early-life exposures [32]. The slight reduction in *Clostridium* observed in the control group, compared with the increase noted in the supplemented group, further supports the hypothesis that maternal probiotic supplementation may promote a broader microbial diversification, which has been generally associated with healthy gut maturation [33].

Overall, these results suggest that maternal supplementation with *L. reuteri* SGL 01 is associated with modifications in neonatal gut colonization patterns, particularly through increases in bacterial genera involved in metabolic and immunological maturation. These results complement existing literature demonstrating the capacity of *L. reuteri* strains to influence the dynamics of the microbial community in neonatal settings [15] and further support the potential role of maternal probiotic intake during lactation in fostering the development of the early-life microbiota [34].

Our findings indicate that maternal probiotic supplementation may benefit full-term, exclusively breastfed infants by promoting increases in *Bifidobacteria* and *Lactobacilli* spp., suggesting a supportive role alongside breastfeeding in shaping a healthy early gut microbiota [22]. Despite these promising preliminary findings, the study presents several limitations that need to be acknowledged. First, the small sample size, further affected by the high dropout rates, has likely reduced the statistical power to detect between-group differences and subtle within-group microbial changes, particularly in breast milk samples, limiting our ability to detect small-to-medium effects and increasing the risk of type II error. In particular, the absence of significant between-group differences may reflect insufficient statistical power rather than the absence of a biological effect. Second, the open-label design may also introduce potential biases; hence, a double-blind, placebo-controlled design is warranted in future validation studies. Furthermore, the use of qPCR restricted the analysis to selected bacterial taxa, precluding a comprehensive assessment of microbial diversity and functional capacity [35]. Future studies incorporating shotgun metagenomics or 16S rRNA sequencing would allow a more detailed characterization of microbial dynamics and strain-level transmission. Additionally, parallel evaluation of immunological or metabolic mediators in breast milk could help elucidate potential functional mechanisms underlying the observed effects on infant gut microbiota.

Although these preliminary findings require validation in larger cohorts, they suggest potential clinical relevance for maternal *L. reuteri* supplementation in specific neonatal subgroups at increased risk of developing unfavorable intestinal microbiota profiles, such as exclusively formula-fed infants [36], exposed to intrapartum antibiotic prophylaxis [37] or delivered by cesarean section [38]. In particular, cesarean section has consistently been associated with reduced abundances of *Bifidobacterium*, *Bacteroides* and *Escherichia* spp., along with increased prevalence of potentially pathogenic taxa in the neonatal gut [38,39]. Given that maternal supplementation with *L. reuteri* SGL 01 was associated with improved neonatal gut colonization in vaginally delivered infants despite detectable changes in breast milk microbiota, targeted studies are warranted. Specifically, future investigations should address whether supplementation in mothers of cesarean-born infants can mitigate the microbial deficits related to this mode of delivery and support a more physiological gut colonization in newborns.

## 5. Conclusions

Maternal supplementation with *Lactobacillus reuteri* SGL 01 during the first post-partum month has been associated with significant within-group increases in specific neonatal gut taxa, including *Lactobacillus* spp. and *Bifidobacterium* spp., which play a crucial role in early-life health. While these preliminary results may suggest a potential indirect maternal-to-infant effect, further research in larger, adequately powered cohorts is strictly required to confirm these observations, especially in populations characterized by unfavorable early microbiota profiles such as those delivered by cesarean section or exposed to intrapartum antibiotic prophylaxis.

## Figures and Tables

**Table 1:** Primer sequences and qPCR conditions used for the study assays.

Target Microorganisms	Primer Sequences (5′-3′)	Amplicon Length (bp)	Annealing Temperature
*Bifidobacterium* spp.			
BifTOT-F	TCGCGTCYGGTGTGAAAG	243	55 °C
BifTOT-R	CCACATCCAGCRTCCAC		
*Lactobacillus* spp.			
Lac-F	GCAGCAGTAGGGAATCTTCCA	349	60 °C
Lac-R	GCATTYCACCGCTACACATG		
*Bacteroides fragilis* group			
Bfra-F	CGGAGGATCCGAGCGTTA	92	58 °C
Bfra-R	CCGCAAACTTTCACAACTGACTTA		
*Clostridium* spp.			
CI-F1	TACCHRAGGAGGAAGCCAC	232	52 °C
CI-F2	GTTCTTCCTAATCTCTACGCAT		

**Table 2:** Protocols for qPCR and concentrations of the primers used for the study assays.

Target Primers	Initial Denaturation	Denaturation	Annealing	Cycles	Fw nM	Rev nM
*Bifidobacterium* spp. BifTOT F/BifTOT-R	95 °C, 20 s	95 °C, 30 s	60 °C, 30 s	40	200	300
*Lactobacillus* spp. LAC-F/LAC-R	95 °C, 20 s	95 °C, 30 s	60 °C, 30 s	40	200	200
*Bacteroides fragilis* group Bfra-F/Bfra-R	95 °C, 20 s	95 °C, 30 s	60 °C, 30 s	40	300	300
*Clostridium* cluster I CI-F1/CI-F2	95 °C, 20 s	95 °C, 30 s	60 °C, 30 s	40	200	200

**Table 3:** Median maternal age, gestational age at birth and neonatal birth weight in the supplemented group and in the control group. Abbreviations: BMI, body mass index; GA, gestational age; IQR, interquartile range.

	Supplemented Group (n = 15)	Control Group (n = 12)	*p*-Value
Maternal age (years), median (IQR)	34 (31–37)	36.5 (34–38.5)	0.130
Smoking during pregnancy, n (%)	0 (0)	1 (8)	0.444
Pre-pregnancy BMI, median (IQR)	21 (20–23.5)	20 (19–21)	0.183
BMI at birth, median (IQR)	26 (23–26.7)	25 (24.5–27)	0.936
GA at birth (weeks), median (IQR)	40 (39–40.5)	39.85 (39–40.5)	1.000
Neonatal birth weight (g), median (IQR)	3325 (3145–3410)	3400 (3193–3663)	1.000

**Table 4:** Concentrations of *Bifidobacteria* and *Lactobacilli* in milk samples from supplemented (n = 15) and control mothers (n = 12) at baseline (T0) and 1 month (T1). Abbreviations: CFU, colony-forming units; IQR, interquartile range.

Maternal Milk Samples	*Bifidobacterium* spp. (Log^10^ CFU/mL)		*p*-Value	*Lactobacillus* spp. (Log^10^ CFU/mL)		*p*-Value
	T0	T1		T0	T1	
Supplemented, median (IQR)	3.45 (2.84–3.68)	3.78 (3.07–4.19)	0.125	5.58 (4.02–6.12)	5.18 (3.47–6.38)	0.629
Controls, median (IQR)	3.78 (3.06–4.66)	3.38 (2.46–4.72)	0.272	5.77 (4.15–6.06)	5.49 (4.76–6.03)	0.754

**Table 5:** Concentrations of *Bifidobacteria* and *Lactobacilli* in fecal samples from the offspring of supplemented (n = 15) and control mothers (n = 12) at baseline (T0) and 1 month (T1). Abbreviations: CFU, colony-forming units; IQR, interquartile range.

Neonatal Fecal Samples	*Bifidobacterium* spp. (Log^10^ CFU/mL)		*p*-Value	*Lactobacillus* spp. (Log^10^ CFU/mL)		*p*-Value
	T0	T1		T0	T1	
Supplemented, median (IQR)	5.69 (5.22–6.23)	7.33 (6.71–8.20)	<0.001	5.09 (4.07–7.46)	7.05 (5.74–7.41)	0.029
Controls, median (IQR)	5.97 (5.19–6.48)	6.98 (5.74–7.44)	0.097	5.47 (5.04–6.89)	7.00 (5.86–7.71)	0.123

**Table 6:** Concentrations of *Clostridium* spp. and *Bacteroides* in fecal samples from the offspring of supplemented (n = 14) and control mothers (n = 7) at baseline (T0) and 1 month (T1). Abbreviations: CFU, colony-forming units; IQR, interquartile range.

Neonatal Fecal Samples	*Clostridium* spp. (Log^10^ CFU/mL)		*p*-Value	*Bacteroides* spp. (Log^10^ CFU/mL)		*p*-Value
	T0	T1		T0	T1	
Supplemented, median (IQR)	2.48 (2.00–4.13)	3.57 (2.22–4.63)	0.003	8.25 (4.66–8.74)	8.16 (4.66–9.10)	0.615
Controls, median (IQR)	3.72 (2.38–5.07)	2.66 (2.21–3.54)	0.046	6.77 (4.55–8.54)	6.06 (3.17–9.14)	0.207

## Data Availability

The study data are available upon reasonable request from the corresponding author.

## Abbreviations

The following abbreviations are used in this manuscript:

BMI	Body mass index
CFU	Colony-forming units (CFU)
GA	Gestational age
IQR	Interquartile range
